# Parental acceptance of pediatric behavior guidance techniques: a cross-sectional study from Egypt

**DOI:** 10.1186/s12903-026-07954-y

**Published:** 2026-03-25

**Authors:** Rodaina H. Helmy, Nour Ammar, May M. Adham, Karin ML. Dowidar, Marwa Baraka

**Affiliations:** Pediatric Dentistry and Dental Public Health Department,Faculty of Dentistry, Alexandria University, Champollion St., El Azarita, Alexandria, Egypt

**Keywords:** Dental caries, Pediatric dentistry, Health survey, Sedation, General anesthesia

## Abstract

**Background:**

Effective behavior guidance in pediatric dentistry depends on both clinical technique and effective communication with parents. Understanding which techniques parents prefer, and how these preferences relate to demographics and dental anxiety, is essential for guiding clinician-parent and supporting informed decision-making.

**Objective:**

To assess the acceptance of twelve behavior-guidance techniques (BGTs) among Egyptian parents of younger (3–6 years) and older (7–12 years) children. Additionally, the study aimed to examine whether parental acceptance is influenced by reported levels of dental anxiety and previous dental experiences.

**Materials and methods:**

Two hundred twenty two parents of children aged 3–12 years scheduled for dental treatments watched video demonstrations of twelve BGTs. The basic guidance techniques included tell-show-do, pre-visit imagery, audio distraction, audiovisual distraction, virtual reality, enhanced control, modeling, social and non-social positive reinforcement, and voice control. Advanced guidance techniques included active protective stabilization and general anesthesia. Parents rated their acceptance 0–5, provided demographic information, and completed the Arabic Modified Dental Anxiety Scale (MDAS). Mann–Whitney U tests were used to compare acceptance scores between two age groups (3–6 and 7–12 years).

**Results:**

The average MDAS score was 13.84 (± 5.35). The mean age was 4.68 (± 1.16) years in the 3–6 group and 9.03 (± 1.61) years in the 7–12 group. Non-social positive reinforcement received the highest acceptance (mean = 4.79), followed by social reinforcement (4.42) and tell-show-do (4.32). Conversely, active protective stabilization (2.77), general anesthesia (2.80), and voice control (2.95) were least accepted. A weak positive correlation (*r* = 0.23, *p* = 0.01) existed between MDAS scores and non-social positive reinforcement acceptance.

**Conclusions:**

Egyptian parents’ acceptance of BGTs varied by child age, socioeconomic status, anxiety, and dental history, with non-social positive reinforcement most favored and active protective stabilization and general anesthesia least preferred. These findings highlight the need for clear, tailored communication when discussing behavior guidance options, particularly for more restrictive techniques.

**Clinical Relevance:**

Understanding parental preferences for BGTs is essential for pediatric dentists to tailor their approaches effectively. This personalized strategy can enhance treatment acceptance, create a more supportive environment for children, and ultimately improve clinical outcomes.

**Supplementary Information:**

The online version contains supplementary material available at 10.1186/s12903-026-07954-y.

## Introduction

In paediatric dentistry, behaviour management is fundamentally a process of communication, in which dentists, children and parents co‑construct meaning around dental guidance and procedures, and in which parents’ own dental anxiety and communicative responses can shape how children perceive and cope with treatment [1]. A pediatric dentist’s armamentarium encompasses a wide range of techniques, each tailored to manage specific behaviors exhibited by the patient during their visit [1]. Effective behavior guidance is essential not only to safeguard optimal clinical outcomes, but also to ensure a positive psychological experience for both the child and the parent [1, 2]. Comprehensive dental guidance is heavily influenced by the parent or caregiver [3]. Parents play a pivotal role in the success of their child’s treatment, being responsible for ensuring proper daily oral hygiene and making healthcare-related decisions [3, 4]. Given that recent research shows that parental dental anxiety can negatively impact a child’s dental behavior [4–6], and that a recent meta-analysis concluded that an estimated 15.3% of adults suffer from dental fear and anxiety [7], addressing parental anxiety and fear is of paramount importance to safeguard the treatment success of pediatric patients.

Various behavior-guidance techniques (BGTs) have been extensively described in the literature [2]. They are all communicative techniques that rely on how information is framed, how instructions are delivered, and how empathy is conveyed to the child. Nonetheless, it has been shown that parental acceptance of the different techniques and their dental anxiety can influence the clinical applicability of these techniques [4–6, 8–10]. Parental dental anxiety not only promotes the child’s dental fear [5, 6, 11], but could also actively shape parents’ preference for specific techniques [4, 12, 13]. Previous research has explored parental preference for BGTs [12, 14–16]. In general, studies conclude that ‘tell-show-do’ is the most widely accepted technique; while conscious sedation, physical restraint, hand-over-mouth, and general anesthesia were among the least favorable [8, 9, 12, 14, 15, 17, 18]. However, with a focus on parental dental anxiety, studies show that parents with a history of adverse dental experiences exhibit a preference for their child’s treatment under general anesthesia over any form of restraint [12] or non-pharmacological behaviour guidance [13]. Conversely, another study concluded that parental anxiety is not associated with their acceptance of specific techniques [10]. This highlights the importance of understanding parental BGT preference, and particularly, in relation to their dental anxiety levels.

Aside from the influence of dental anxiety, studies also highlight that parental attitudes [3] and societal norms [11, 18, 19] are among the main determinants of children’s dental anxiety [5, 6] and oral health status [11]. Country-specific investigations from various nations (e.g., Greece [12], Spain [14], Germany [18], Pakistan [13], among others) have documented this variability. To that extent, there remains a knowledge gap regarding how Egyptian parents perceive and accept various BGTs.

Although many of the techniques described in the literature are routinely employed by pediatric dentists in Egypt, systematic investigations into their use and acceptance are scarce [20, 21]. Moreover, there is a distinct gap in the literature concerning parental preferences for these techniques, especially studies that explicitly account for the influence of parental dental anxiety on such preferences. Understanding these preferences is critical in reinforcing the use of the most effective and culturally accepted methods for different patient groups. Hence, this study aimed to address this gap in the literature. The aims of the present study are: first, to assess the acceptance and preferences of Egyptian parents of younger (3–6 years) and older (7–12 years) children for twelve different behavior-management techniques; and second, to examine whether parental acceptance is influenced by their reported levels of dental anxiety and previous dental experiences. The null hypothesis posited that there is no relationship between parents’ dental anxiety and/or dental experience and their acceptance of different BGTs.

## Methods

### Study Design and Ethical Considerations

This observational cross-sectional study is reported following the Strengthening the Reporting of Observational Studies in Epidemiology (STROBE) Statement [22]. The study took place at the Department of Pediatric Dentistry and Dental Public Health at the Faculty of Dentistry, Alexandria University, from June to August 2024. It was conducted in accordance with the Declaration of Helsinki and received the approval of the Research Ethics Committee from the Faculty of Dentistry (IORG0008839 #0907–04/2024). Both verbal and written informed consent were obtained from all participants. To ensure confidentiality and anonymity, all questionnaires were coded without personal identifiers, ensuring that responses could not be traced back to individual participants.

### Sampling and Eligibility criteria

Participants were recruited using a convenience sampling technique, including all eligible parents attending the Pediatric Dentistry Department at the Faculty of Dentistry, Alexandria University, during the study period. Eligible participants were parents or legal guardians of 3-12-year-old children receiving dental treatment at the pediatric dentistry department. Children were categorized into two age groups: preschoolers (3–6 years) and school-aged children (7–12 years). The sample included 114 (51.4%) female and 108 (48.6%) male children. The completion of the written informed consent form was a prerequisite for participation. The eligibility criteria specifically included parents of healthy patients (American Society of Anesthesiologists (ASA) class I) to minimize potential biases that could arise from pre-existing conditions affecting the child’s behavior or dental experience. The exclusion criteria were parents of children receiving pharmacological treatment for a chronic disorder or parents of children with special health care needs.

### Sample size estimation

The sample size calculation was performed using the online calculator at https://www.calculator.net/sample-size-calculator.html based on a 95% confidence level and 5% marginal error. Based on a previous study, the percentage of acceptance of the different BGTs ranged from 14.7% for the physical restraint; which was the least accepted method, to 91.5% for the tell-show-do; which had the highest acceptance level [15]. The minimum required sample size was calculated to be 192 patients [23]; this was increased by 10% to make up for the non-response participants. The final sample size was 211.

### Development of the study video

Due to copyright restrictions, cultural differences, and language barriers, it was not feasible to use a copy of the videos used in previous similar studies [8, 9, 14, 17]. As a result, a series of videos comparable to those created in the previous studies was custom-created for use in the present study. The videos featured an introduction to each behavior-management technique in the national language –Arabic–, with clear explanations provided for each technique individually, that aligned with the American Academy of Pediatric Dentistry (AAPD) guidelines. Healthy children were selected to film the BGTs in the new videos after obtaining written informed consent from their parents or legal guardians and verbal assent from the children. The twelve BGTs demonstrated in the videos were classified based on AAPD guidelines into Basic Behavior Guidance and Advanced Behavior Guidance as follows:

- *Basic Behavior Techniques*
  *• Tell-Show-Do*: Verbal explanations of a dental tool (air-water syringe) were recorded, followed by demonstrations on the child's hands and then using it intraorally.
  *• Pre-Visit Imagery*: Positive images of the clinic setting (dental chair, child-friendly decorations) were shown to prepare children before appointments.
  *• Audio Distraction*: A child was recorded while wearing headphones and listening to any song of his choice.
  *• Audiovisual (AV)Distraction*: A child was recorded during a dental visit while watching a cartoon film on a mobile screen.
  *• Visual Reality (VR)*: A child was recorded during a dental visit while wearing VR glasses.
  *• Enhanced Control*: Children signaled discomfort by raising their hands, prompting the dentist to stop.
  *• Non-Social Positive Reinforcement*: Children were given gifts for desired behaviors at the end of the visit. Gifts were age-appropriate items such as toys and stationery, selected to be suitable for children of varying ages and genders.
  *• Social Positive Reinforcement*: Praising and affirmative gestures were used to reward cooperative behaviors.
  *• Modeling*: A child observed another child enjoying his dental treatment.
  *• Voice Control*: Altering voice tone was done to direct an uncooperative patient's behavior.
- *Advanced Behavior Techniques*
  - *Active Protective Stabilization*: Physical limitation of an uncooperative child by his parent or legal guardian. Parents in the video were shown the professional holding techniques to ensure the child's safety without the use of mechanical restraining devices.
  - *General Anesthesia*: A child was being checked for eligibility, followed by the induction of anesthetic drugs, treatment, and subsequent arousal.

The content of the videos underwent a rigorous validation process to confirm their accuracy, relevance, and appropriateness for the intended educational purpose. The videos were developed in collaboration with pediatric dentistry experts to ensure that the content accurately represented the BGTs. Face validity assessment was conducted by asking a panel of nine experienced pediatric dentists, each with more than 15 years of experience, to review the videos and evaluate the clarity and appropriateness of the techniques depicted. The content validity score of the videos was 0.94, indicating a high level of agreement among the reviewers regarding the relevance and quality of the videos. They provided constructive feedback on the content, presentation style, and educational value. Revisions were made based on their recommendations to enhance the videos’ effectiveness and ensure they met educational standards. This expert review, with a content validity score of 0.94, provided adequate validation that the videos were appropriate and reflective of real-world pediatric dental BGTs.

To assess the understanding and acceptance of the techniques presented, the videos were pilot tested with a small group of parents. During this pilot phase, parents were asked to provide feedback on the clarity of the visuals and the effectiveness of the voice-over narration. Parents in the pilot group confirmed that the total duration was acceptable and did not result in fatigue. This feedback was systematically analyzed to identify any areas of confusion or misinterpretation, allowing for further refinements. All videos were standardized in terms of duration and production quality. Each video lasted approximately 20–30 s. In addition, they were all recorded in the same setting with the same operator performing all BGTs to ensure consistency across presentations. Each video was carefully timed to ensure equal duration, and a consistent voice-over narration was employed to provide uniform information throughout. This approach aimed to minimize bias and maintain focus on the techniques rather than variations in presentation.

During the main study, the videos were presented in a randomized order to each participant to avoid any sequence-related bias and ensure that respondent fatigue did not systematically affect specific techniques. Randomization was achieved using a random number generator to assign the order of video presentation for each participant, ensuring a fair evaluation of each BGT [12]. The BGTs included (in no specific order): tell-show-do, pre-visit imagery, audio distraction, audiovisual distraction, visual reality, enhanced control, modeling, non-social positive reinforcement, social positive reinforcement, voice control, active protective stabilization, and treatment under general anesthesia [2].

The questionnaire used to assess parental acceptance was also carefully validated. It was adapted from previously validated instruments [10, 12]. To ensure content validity, the same panel of nine pediatric dentistry experts reviewed the items for clarity, relevance, and appropriateness. The internal consistency of the questionnaire was assessed using Cronbach’s alpha, which yielded a value of 0.85, indicating good reliability.

### Study outcomes

Our study’s primary outcome was parental acceptance of various dental techniques through a structured approach. Acceptance levels were measured using a 5-point Likert scale, where a rating of 0 indicated complete opposition to the technique, and a rating of 5 signified full acceptance. Parents watched alone a video featuring these techniques before their child’s dental visit and had 10 s to rate each technique [12].

After completing their ratings, parents filled out an anonymous questionnaire adapted from previous research [14, 17], which collected demographic information, including gender, age, family income, and educational level (ranging from no formal education, primary-level education (1–6 years), middle school-level education (7–9 years), secondary-level education (10–12 years), and university-level education). Additionally, the questionnaire assessed previous dental experiences for both the parents (self-reported) and the children (obtained via parental report and confirmed by the children themselves when engaged). Participants were asked to categorize these past experiences based on subjective perception as: ‘positive’ (satisfactory/non-traumatic), ‘negative’ (traumatic/unpleasant), or ‘no experience’ (never visited a dentist). At the end of the questionnaire, parents were asked to indicate their preference between general anesthesia and one of the techniques (active protective stabilization or voice control) if their child was uncooperative during treatment.

The second outcome of the study focused on assessing the dental anxiety levels of the parents. For this purpose, the Arabic version of the modified dental anxiety scale (MDAS) was employed [24]. It consists of five items, each designed to assess different aspects of dental anxiety. Parents rate their anxiety on a scale from 1 (not anxious) to 5 (extremely anxious), resulting in a total score ranging from 5 to 25. Higher scores indicate greater levels of anxiety. Previous studies have validated this scale, demonstrating its adequate internal consistency and reliability, which allows for effective assessment of dental anxiety among Arabic-speaking populations [24–28].

### Statistical analysis

Data were analyzed using both descriptive and inferential statistics. Descriptive statistics were used to summarize the demographic characteristics of the participants, including the age of the children (mean ± standard deviation) and parental income (mean ± standard deviation). Categorical variables, such as parental education (father’s and mother’s), previous dental experience of the parents, and previous dental experience of the children, were presented as frequencies and percentages. A pie chart and bar graph were used to visually represent the distribution and preference of parents for different BGTs.

To assess parental acceptance of the different BGTs, mean scores were calculated for each technique. Normality tests (Shapiro-Wilk) were performed for the acceptance scores. Given that the data were not normally distributed, the Kruskal-Wallis H test was used to compare the acceptance scores across the different techniques. Post-hoc pairwise comparisons with Bonferroni correction were conducted to determine which techniques differed significantly in terms of parental acceptance.

Participants were stratified into two age groups: preschool children (3–6 years) and school-aged children (7–12 years). Group comparisons were made using the Mann–Whitney U test to assess differences in acceptance between the two strata. Spearman’s rank correlation coefficient was calculated to assess the relationship between parental acceptance of each BGT and the modified dental anxiety scale. Additionally, multiple linear regression analyses were performed to identify significant predictors of parental acceptance for each BGT. Independent variables include the child’s age, parental gender, educational level of the mother, monthly income, and parental dental anxiety (MDAS score). A significance level of 0.05 was applied to all statistical tests. All statistical analyses were conducted using SPSS version 26.0, and results are reported with p-values and 95% confidence intervals, where applicable.

## Results

**Fig. 1:**
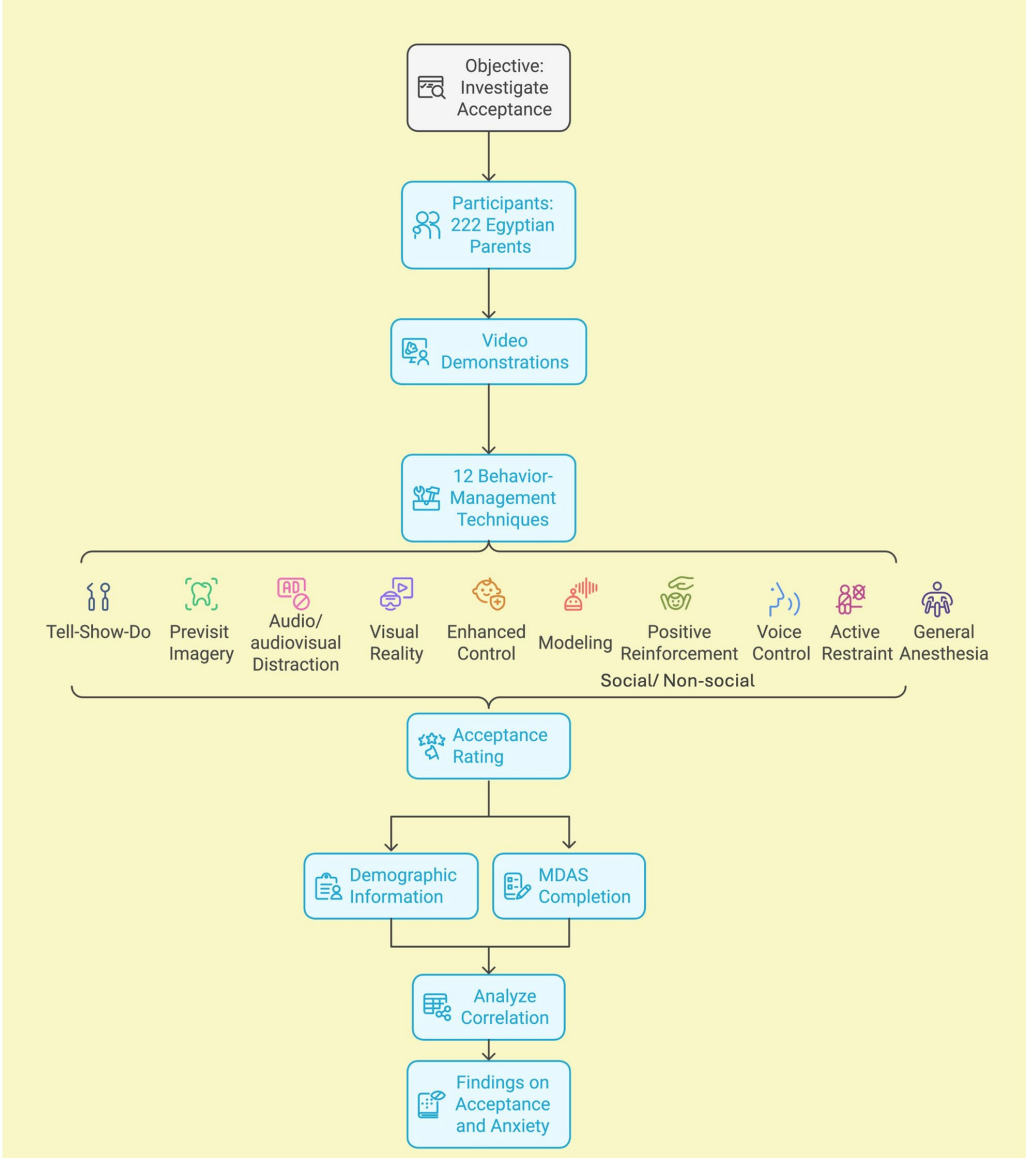
Study flow diagram

A total of 222 parents participated in the study. Of these, 93 were parents of preschool-aged children (3–6 years, 41.9%; children’s mean age ± SD = 4.68 ± 1.16 years) and 129 were parents of school-aged children (7–12 years, 58.1%; children’s mean age ± SD = 9.03 ± 1.61 years). In terms of gender distribution, there were 114 (51.4%) female and 108 (48.6%) male children. Most participants were mothers (67.1%), followed by fathers (15.3%) and other relatives (17.6%). A majority of both mothers (79.7%) and fathers (70.3%) held university degrees. Regarding previous dental experiences, 75.2% of parents reported positive experiences, and 67.5% of children had positive prior dental experiences, while 9.5% of children had never visited a dentist (Table 1). The study flow chart is shown in Fig. 1.

**Table 1:** Demographic characteristics of the study sample

	mean (SD)
Age (years)	7.21(2.59)
Preschoolers (3–6 years)	4.68(1.16)
School-aged children (7–12 years)	9.03(1.61)
Parental Income (EGP)	10002.28(14871.11)
	**n(%)**
Age groups (years)	
Preschoolers (3–6 years)	93(41.9)
School-aged children (7–12 years)	129(58.1)
Gender	
Male	108(48.6)
Female	114(51.4)
Parent	
Mother	149(67.1)
Father	34(15.3)
Other relatives	39(17.6)
Mother’s education	
No education	1(0.5)
Primary education	4(1.8)
Middle education	22(9.9)
Secondary education	8(3.6)
University education	177(79.7)
Don’t know	10(4.5%)
Father’s education	
No education	2(0.9)
Primary education	14(6.3)
Middle education	30(13.5)
Secondary education	3(1.4)
University education	156(70.3)
Don’t know	14(6.3)
Previous parent’s dental experience	
no experience	19(8.6)
negative	36(16.2)
positive	167(75.2)
Previous child’s dental experience	
no experience	21(9.5)
negative	51(23)
positive	150(67.5)

**Fig. 2:**
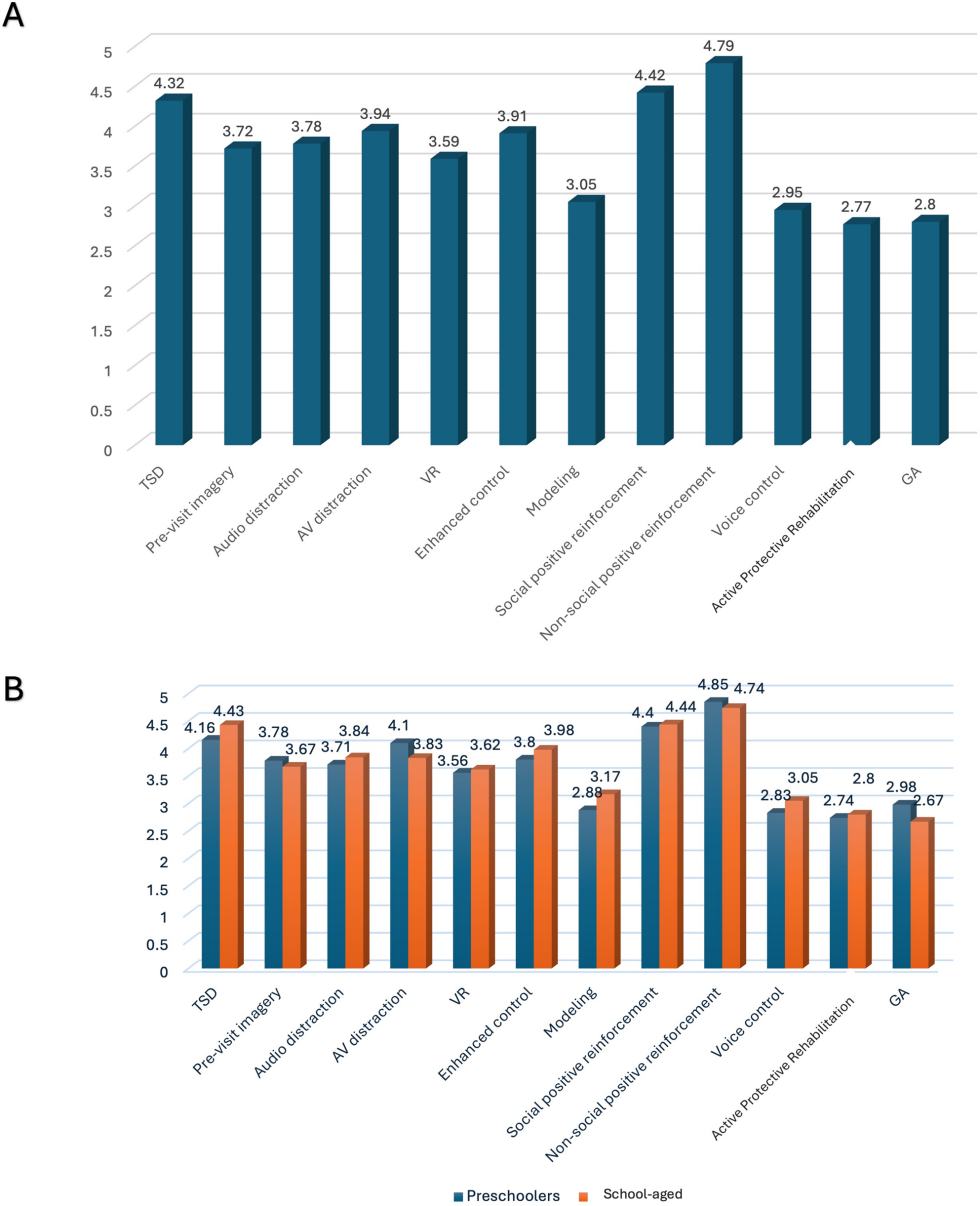
**A** Mean values of parental acceptance of different behaviour guidance techniques. **B **Mean values of parental acceptance of different behaviour guidance techniques by age group

Parental acceptance varied across the twelve BGTs. Non-social positive reinforcement received the highest acceptance (mean = 4.79), followed by social reinforcement (4.42) and Tell-Show-Do (4.32), while active protective stabilization (2.77), general anesthesia (2.80), and voice control (2.95) were the least accepted (Fig. 2A).

Comparisons between age groups showed that Tell-Show-Do was significantly more accepted for school-aged children (mean = 4.43) than preschoolers (mean = 4.16, *p* = 0.006), whereas audiovisual distraction was significantly more accepted for preschoolers (mean = 4.1) than older children (mean = 3.83, *p* = 0.014). No statistically significant differences were observed between age groups for the remaining BGTs (*p* > 0.05) (Table 2; Fig. 2B).

**Table 2:** Parental acceptance of behaviour guidance techniques by age group

	Preschoolers		School-aged children		
	Mean	SD	Mean	SD	*P* vale
Tell-Show-Do	4.16	0.811	4.43	0.716	0.006*
Pre-visit imagery	3.78	0.858	3.67	0.812	0.265
Audio distraction	3.71	0.854	3.84	0.9	0.18
AV distraction	4.1	0.99	3.83	0.977	0.014*
VR	3.56	1.058	3.62	1.077	0.589
Enhanced control	3.8	0.828	3.98	0.515	0.098
Modeling	2.88	1.121	3.17	1.024	0.051
Social positive reinforcement	4.4	0.628	4.44	0.558	0.746
Non-social reinforcement	4.85	0.36	4.74	0.438	0.059
Voice control	2.83	1.1	3.05	1.211	0.182
Active protective stabilization	2.74	1.112	2.8	1.319	0.905
GA	2.98	1.251	2.67	1.246	0.072

*Significant (*p* ≤ 0.05)

Parental dental anxiety, measured by MDAS, averaged 13.84 ± 5.35, with a weak positive correlation between MDAS and acceptance of non-social positive reinforcement (*r* = 0.23, *p* = 0.01), indicating that parents with higher anxiety tended to favor this technique more. An intermediate negative correlation was observed with VR acceptance (*r* = − 0.34, *p* < 0.01), indicating that higher parental anxiety was associated with lower acceptance of VR. No significant correlations were observed for other techniques. Income was positively correlated with Tell-Show-Do (*r* = 0.33, *p* < 0.01), social positive reinforcement (*r* = 0.35, *p* < 0.01), and non-social positive reinforcement (*r* = 0.22, *p* = 0.01), indicating that parents with higher income were more accepting of these techniques. Conversely, negative correlations were found for pre-visit imagery (*r* = − 0.27, *p* < 0.01), active protective stabilization (*r* = − 0.31, *p* < 0.01), and voice control (*r* = − 0.24, *p* < 0.001), suggesting that higher-income parents were less accepting of these techniques. Regarding management of noncooperative children, parents were nearly evenly split, with 51% preferring active protective stabilization and 49% preferring general anesthesia.

The impact of demographic characteristics on parental acceptance revealed that fathers showed higher acceptance of audio techniques (mean = 4.21) compared to mothers (3.68), while other legal guardians preferred audiovisual distraction (4.31) over both mothers (3.85) and fathers (3.94). Mothers favored non-social positive reinforcement (4.77) and modeling (4.0), particularly those with secondary education, whereas fathers with university education reported high acceptance of Tell-Show-Do (4.34), social (4.44), and non-social positive reinforcement (4.83). Similarly, mothers with university education showed greater acceptance of the same three BGTs, mirroring the pattern observed among fathers. Previous experiences also significantly influenced acceptance. Parents of children with positive dental experiences reported higher acceptance for modeling (3.29), pre-visit imagery (3.80), audiovisual distraction (4.05), enhanced control (4.07), and non-social positive reinforcement (4.83), while parents of children with negative experiences had lower acceptance for pre-visit imagery (3.41), audiovisual distraction (3.43), enhanced control (3.53), modeling (2.69), voice control (2.12), and active protective stabilization (2.10). Non-social positive reinforcement remained highly accepted in the latter group (4.73) (Table 3).

**Table 3:** Effect of demographic characteristics on parental acceptance of different behaviour guidance techniques

	TSD		Pre-visit imagery		Audio distraction		AV distraction		VR		Enhanced control		Modeling		Social positive reinforcement		Non-social positive reinforcement		Voice control		Active protective stabilization		GA	
	mean	*P* value	mean	*P* value	mean	*P* value	mean	*P* value	mean	*P* value	mean	*P* value	mean	*P* value	mean	*P* value	mean	*P* value	mean	*P* value	mean	*P* value	mean	*P* value
Parent		0.927		0.068		0.006*		0.008*		0.891		0.002*		0.935		0.5		0.015*		0.67		0.902		0.09
Mother	4.3		3.76		3.68^a^		3.85^a^		3.55		3.99^a^		3.05		4.48		4.77^a^		2.93		2.78		2.85	
Father	4.29		3.85		4.21^b^		3.94		3.68		3.47^b^		3.09		4.47		4.97^b^		3.12		2.68		2.35	
Other relatives	4.38		3.46		3.79		4.31^b^		3.69		3.97^a^		3		4.18		4.72^a^		2.92		2.85		3	
Mother’s education		0.017*		0.658		0.562		0.759		0.184		0.624		0.002*		0.196		0.009*		0.24		0.305		0.129
No education	4		4		4		4		4		4		2		4		4		2		4		4	
Primary education	4.25		3.25		3.75		3.5		4.25		4		3.5		4		4.25^a^		3.5		3.5		3.5	
Middle education	4.14		3.55		3.64		4.05		3.95		3.73		2.5^a^		4.55		4.77		3.27		3		2.68	
Secondary education	4.75^a^		4		4.25		4.25		3.88		4		4.25^b^		4.63		5^b^		3.25		3.25		2.13	
University education	4.34		3.73		3.77		3.92		3.52		3.92		3.03^a^		4.42		4.81		2.86		2.68		2.78	
Don’t know	4^b^		3.9		4		4.1		3.6		3.9		3.5		4.2		4.6		3.5		3.1		3.5	
Father’s education		0.051		0.001*		0.353		0.094		0.051		0.674		0.227		0.362		0.008*		0.484		0.513		0.003*
No education	4		3		4		5		2		4		2		5		5		4		4		1^a^	
Primary education	4.14		3.71		3.71		3.79		3.79		4		2.79		4.5		4.5^a^		3.29		3.14		2.64	
Middle education	4.23		3.93		3.9		4.17		3.47		3.87		3		4.37		4.73		3.03		2.8		3.2	
Secondary education	5		3.33		2.67		2.67		2		4		4		4		4.33		2.67		2.67		4^b^	
University education	4.34		3.63^a^		3.81		3.92		3.63		3.93		3.05		4.44		4.83^b^		2.94		2.74		2.65	
Don’t know	4.36		4.43^b^		3.5		3.93		3.93		3.57		3.43		4.43		4.93		2.57		2.43		3.64^b^	
Previous parent’s dental experience		0.248		0.055		0.033*		0.001*		0.043*		0.001*		0.07		0.134		0.041*		< 0.01*		0.043*		0.678
no experience	4.21		3.95		3.84		4.37^a^		3.63		4.05^a^		2.84		4.53		4.79		3.74^a^		2.79		2.58	
negative	4.14		3.39		3.47^a^		3.39^b^		3.17^a^		3.44^b^		2.72		4.53		4.94^a^		2.19^b^		2.31^a^		2.69	
positive	4.37		3.77		3.84^b^		4.01^a^		3.68^b^		3.99^a^		3.14		4.39		4.75^b^		3.03^c^		2.87^b^		2.84	
Previous child’s dental experience		0.858		0.013*		0.057		< 0.001*		0.471		< 0.001*		< 0.001*		0.732		0.037*		< 0.001*		< 0.001*		0.371
no experience	4.38		3.9		3.95		4.38^a^		3.62		3.67^a^		2.19^a^		4.24		4.62^a^		3^a^		2.86		3.14	
negative	4.27		3.41^a^		3.55		3.43^b^		3.41		3.53^a^		2.69^a^		4.41		4.73		2.12^b^		2.1^a^		2.82	
positive	4.32		3.8^b^		3.84		4.05^a^		3.65		4.07^b^		3.29^b^		4.45		4.83^b^		3.23^a^		2.99^b^		2.74	

a, b,c Different letters denote significant difference

*Significant(*p* ≤ 0.05)

Multiple linear regression identified several significant predictors of parental acceptance of BGTs. Older child age was associated with higher acceptance of Tell-Show-Do (B = 0.67, *p* = 0.001) and lower acceptance of audiovisual distraction (B = − 0.071, *p* = 0.011). Higher parental dental anxiety was associated with lower acceptance of Tell-Show-Do (B = -0.032, *p* = 0.001) and VR distraction (B=-0.067, *p* < 0.001), but greater acceptance of non-social positive reinforcement (B = 0.016, *p* = 0.001). Higher paternal education predicted greater acceptance of VR distraction (B = 0.209, *p* = 0.004), while higher maternal education predicted lower acceptance of the same technique (B = − 0.274, *p* = 0.005). Positive previous dental experiences in children significantly predicted greater acceptance of enhanced control (B = 0.283, *p* = 0.001), modeling (B = 0.537, *p* < 0.001), social positive reinforcement (B = 0.187, *p* = 0.009), voice control (B = 0.503, *p* < 0.001), non-social positive reinforcement (B = 0.139, *p* = 0.004) and active protective stabilization (B = 0.397, *p* = 0.010) (Table 4).

**Table 4:** Multiple linear regression analysis of predictors of parental acceptance of behaviour guidance techniques

	TSD		Pre-visit imagery		Audio distraction		AV distraction		VR		Enhanced control		Modeling		Social positive reinforcement		Non-social positive reinforcement		Voice control		Active protective stabilization		GA	
	B	*P*	B	*P*	B	*P*	B	*P*	B	*P*	B	*P*	B	*P*	B	*P*	B	*P*	B	*P*	B	*P*	B	*P*
Age	0.67	0.001*	-0.005	0.837	0.001	0.962	-0.071	0.011*	-0.007	0.811	0.023	0.193	0.021	0.466	0.002	0.923	-0.013	0.216	0.031	0.323	0.02	0.546	-0.048	0.177
Total MDAS	-0.032	0.001*	-0.014	0.169	-0.001	0.943	0.009	0.483	-0.067	< 0.001*	-0.002	0.804	0.001	0.954	-0.01	0.156	0.016	0.001*	-0.013	0.378	0.004	0.817	0	0.994
Father’s education	0.66	0.211	0.059	0.302	-0.028	0.663	-0.119	0.096	0.209	0.004*	-0.037	0.428	0.074	0.326	-0.03	0.459	0.027	0.331	-0.097	0.232	-0.096	0.272	0.012	0.897
Mother’s education	0.002	0.978	0.115	0.132	0.056	0.509	0.014	0.885	-0.274	0.005*	0.017	0.785	-0.043	0.665	-0.076	0.158	-0.02	0.577	-0.107	0.319	-0.201	0.084	0.056	0.647
Previous experience (parent)	0.118	0.162	0.039	0.667	0.104	0.31	0.009	0.937	0.139	0.229	0.005	0.943	0.04	0.74	-0.15	0.021*	-0.112	0.011*	-0.227	0.082	0.049	0.725	0.253	0.087
Previous experience (child)	-0.102	0.271	-0.046	0.648	0.03	0.789	0.18	0.149	0.07	0.582	0.283	0.001*	0.537	< 0.001*	0.187	0.009*	0.139	0.004*	0.503	< 0.001*	0.397	0.010*	-0.203	0.208
Gender	0.145	0.158	-0.033	0.764	0.193	0.122	-0.014	0.919	-0.197	0.161	0.009	0.919	0.104	0.474	0.011	0.892	-0.094	0.081	-0.196	0.214	0.062	0.717	0.189	0.292
Relative	0.00	0.982	-0.6	0.007*	0.012	0.632	0.072	0.009*	0.004	0.899	0.014	0.434	0.014	0.635	-0.052	0.001*	-0.003	0.806	0.038	0.223	0.18	0.602	0.012	0.734

*Significant(*p* ≤ 0.05)

## Discussion

Effective communication between parents and dentists is essential for delivering optimal dental care to pediatric patients. Beyond their clinical function, behavior guidance techniques serve as structured communication strategies through which pediatric dentists convey information, reassurance, and expectations to children and their parents. Accordingly, parental acceptance reflects not only treatment preferences, but also perceptions of dentists’ empathy, transparency, and respect for child autonomy. Literature has shown that the BGTs utilized in pediatric dentistry have evolved, influenced by factors such as parental acceptance, legal considerations, ethical dilemmas, and access to certain techniques [2, 18]. Thus, this cross-sectional observational study investigated Egyptian parents’ acceptance of twelve BGTs used in pediatric dentistry and examined the influence of parental dental anxiety, sociodemographic factors, and previous dental experiences. Overall, parents showed greater acceptance of basic communicative techniques, particularly non-social positive reinforcement, social positive reinforcement, and Tell-Show-Do, while advanced or restrictive techniques such as active protective stabilization and general anesthesia were least accepted. These findings highlight the importance of understanding parental expectations when selecting behavior guidance strategies in pediatric dental practice.

Utilizing recorded videos in Arabic, accompanied by Arabic transcripts, effectively conveyed essential information to parents about various pediatric BGTs while considering cultural differences between Egypt and other countries. Notably, using videos in the national language served as a standardized communication medium that bridged the health literacy gap and ensured that technical procedures were accessible to all participants. This approach significantly enhanced parents’ acceptance of these techniques, aligning with the findings of Mirmoeini et al. (2020), which reached similar conclusions regarding the effectiveness of these techniques [29].

In our current study, we examined both traditional and innovative BGTs, specifically twelve methods derived from the American Academy of Pediatric Dentistry’s 2024 guidelines [1]. This approach differs from previous studies that primarily focused on traditional techniques such as Tell-Show-Do, voice control, general anesthesia, physical and active protective stabilizations, nitrous oxide sedation, and the hand-over-mouth technique [1, 12, 27–29]. We excluded nitrous oxide sedation, as it is not utilized in Egypt, and the hand-over-mouth technique, which is contraindicated according to the latest recommendations [1].

The current findings indicate a strong preference for communicative and reinforcement-based techniques, such as Tell-Show-Do and positive reinforcement, while invasive methods like active protective stabilization and general anesthesia were the least accepted. These results align with research by Guinot et al. [8] and Qureshi et al. [9], both of which found communicative approaches to be most favorable among diverse parental groups. However, a notable divergence exists regarding voice control; while Spanish and Portuguese parents in Guinot’s study viewed it favorably, our participants showed significant resistance, highlighting cultural variations in the perception of authority-based techniques. These cross-study comparisons are essential for highlighting the role of cultural context in shaping parental perceptions of behavior guidance techniques, as attitudes toward authority, technology use, and child autonomy vary considerably across populations.

Our study showed that parents of older children were more likely to accept the Tell-Show-Do technique and less likely to accept audiovisual distraction. This preference likely reflects parental expectations of cognitive maturity and a preference for active coping and transparent communication over passive diversion, suggesting that as children age, parents prioritize transparent communication and educational engagement to build long-term dental confidence [30].

Parental dental anxiety significantly influenced the acceptance of specific guidance methods. Higher anxiety levels were associated with an increased preference for non-social positive reinforcement, such as tangible rewards, which parents may view as a necessary comfort strategy. In contrast, highly anxious parents were less accepting of VR, potentially fearing that immersive technology could increase distress or overwhelm the child. This aligns with Felemban et al., who suggested that VR goggles might heighten anxiety by blocking environmental awareness [31]. These findings contrast with Boka et al. [12] and Shukla et al. [10], who reported no correlation between anxiety and technique acceptance, a discrepancy likely rooted in cultural differences regarding technology and dental fear. Notably, regression analysis showed that higher anxiety was also associated with a lower acceptance of Tell-Show-Do; this could suggest that anxious parents may fear that transparently explaining procedures might inadvertently trigger or escalate their child’s distress.

Socioeconomic factors further shaped these preferences, as higher income and education levels were linked to a greater acceptance of communicative techniques; Tell-Show-Do and positive reinforcement- social and non-social. This likely reflects higher health literacy and a preference for child-centered, participatory care. Conversely, intermediate negative correlations were observed between higher income and acceptance of voice control, and active protective stabilization, possibly reflecting a preference for less authoritative or potentially distressing approaches.

Family roles also played a part; fathers favored nonsocial reinforcement but were less accepting of enhanced control, possibly prioritizing outcome-based strategies and financial responsibility. Fathers were more accepting of audio distraction than mothers, who often expressed reservations about screen use, whereas other caregivers demonstrated greater acceptance of audiovisual distraction. However, qualitative research is required to fully explore the motivations behind these gender-based differences.

Previous dental history significantly influenced BGT acceptance, as parents of children with positive past experiences were more receptive to communicative methods, modeling, and social reinforcement. This openness likely reflects a foundation of trust in the clinical process, extending even to advanced techniques like active protective stabilization. Interestingly, previous child experience did not significantly predict parental acceptance of general anesthesia. Nevertheless, parents who rejected protective stabilization also tended to show low acceptance of GA. Although this association was not explained by the regression model, it may reflect a broader parental reluctance toward interventions that substantially limit child autonomy or involve heightened perceived risk. This finding highlights the importance of targeted counseling and shared decision-making when advanced behavior guidance techniques are proposed.

Clinically, these findings emphasize the need for dentists to tailor behavior guidance explanations based on parental anxiety levels, child age, and prior dental experiences. Parents with high dental anxiety and those whose children have had negative past dental experiences may require additional reassurance, and the use of nonsocial positive reinforcement may help improve parental trust and cooperation during treatment planning.

Our study possesses several notable strengths. Firstly, we employed validated videos presented in a randomized order, which minimized bias related to the perceived complexity or severity of BGTs. Additionally, it addresses a significant research gap, as there are very few studies evaluating parental acceptance of such techniques in the Middle East. The existing studies are outdated and do not reflect current parental beliefs, attitudes, or techniques. Importantly, to our knowledge, no prior research has been conducted in Egypt on this topic, highlighting the novelty and importance of our work in understanding parental perceptions within our cultural context.

Despite its insights, this study has several limitations. First, although the Faculty of Dentistry at Alexandria University serves a large, socioeconomically diverse population through both free and paid services, the use of a convenience sample from a single university clinic may limit the generalizability of the findings to the broader Egyptian population. Additionally, the reliance on a 5-point Likert scale introduces potential subjectivity and recall bias. While our analysis accounted for a broad age range (3–12 years) through subgroup modeling, the study was not specifically powered for age-stratified comparisons; thus, these results should be interpreted with caution and validated in future research.

Our findings underscore the importance of aligning clinical practices with parental expectations to enhance the pediatric dental experience. Future research should utilize longitudinal designs and more diverse, representative samples to further investigate the complex relationships between parental acceptance, child behavior, and clinical outcomes.

## Conclusion

Egyptian parents’ acceptance of BGTs varies significantly based on socioeconomic factors, anxiety levels, and previous dental history. While communicative methods and positive reinforcement are highly favored, invasive approaches remain generally unacceptable. Consequently, pediatric dentists should employ individualized communication strategies, tailoring explanations based on child age and offering additional reassurance, through non-social positive reinforcement, to anxious parents and those with negative dental histories.

## Supplementary Information

Supplementary Material 1.

## Data Availability

The datasets used and/or analyzed during the current study are available from the corresponding author upon reasonable request.

## Abbreviations

BGTs: Behavior Guidance Techniques
AV: Audiovisual
VR: Virtual Reality
GA: General Anesthesia
MDAS: Modified dental anxiety scale
