# Supplementary material for: Parental acceptance of pediatric behavior guidance techniques: a cross-sectional study from Egypt
Source: BMC Oral Health. 2026 Mar 25;26:720. doi: 10.1186/s12903-026-07954-y (PMC13104196; doi:10.1186/s12903-026-07954-y)
Supplement: Supplementary file 1 — Supplementary Material 1. [file 12903_2026_7954_MOESM1_ESM.docx]

STROBE Statement—checklist of items that should be included in reports of observational studies

|  | Item No | Recommendation | Page  No |
| --- | --- | --- | --- |
| **Title and abstract** | 1 | (*a*) Indicate the study’s design with a commonly used term in the title or the abstract | Page 1 line 2 & page 2 line 36 |
|  |  | (*b*) Provide in the abstract an informative and balanced summary of what was done and what was found | 2 lines 33-64 |
| Introduction | | | |
| Background/rationale | 2 | Explain the scientific background and rationale for the investigation being reported | 3 & 4 lines 68-112 |
| Objectives | 3 | State specific objectives, including any prespecified hypotheses | 4, lines 107-112 |
| Methods | | | |
| Study design | 4 | Present key elements of study design early in the paper | 4, lines 115-124 |
| Setting | 5 | Describe the setting, locations, and relevant dates, including periods of recruitment, exposure, follow-up, and data collection | 4, lines 117-124 |
| Participants | 6 | (*a*) *Cohort study*—Give the eligibility criteria, and the sources and methods of selection of participants. Describe methods of follow-up  *Case-control study*—Give the eligibility criteria, and the sources and methods of case ascertainment and control selection. Give the rationale for the choice of cases and controls  *Cross-sectional study*—Give the eligibility criteria, and the sources and methods of selection of participants | 4 & 5, lines 126-138 |
|  |  | (*b*) *Cohort study*—For matched studies, give matching criteria and number of exposed and unexposed  *Case-control study*—For matched studies, give matching criteria and the number of controls per case |  |
| Variables | 7 | Clearly define all outcomes, exposures, predictors, potential confounders, and effect modifiers. Give diagnostic criteria, if applicable | 8, lines 225-250 |
| Data sources/ measurement | 8* | For each variable of interest, give sources of data and details of methods of assessment (measurement). Describe comparability of assessment methods if there is more than one group | 5-7, lines 147-224 |
| Bias | 9 | Describe any efforts to address potential sources of bias | 3-5 |
| Study size | 10 | Explain how the study size was arrived at | 5, lines 140-146 |
| Quantitative variables | 11 | Explain how quantitative variables were handled in the analyses. If applicable, describe which groupings were chosen and why | 8 & 9, lines 251-274 |
| Statistical methods | 12 | (*a*) Describe all statistical methods, including those used to control for confounding | 8 & 9, lines 251-274 |
|  |  | (*b*) Describe any methods used to examine subgroups and interactions | 8 & 9, lines 251-274 |
|  |  | (*c*) Explain how missing data were addressed | 58 & 9, lines 251-274 |
|  |  | (*d*) *Cohort study*—If applicable, explain how loss to follow-up was addressed  *Case-control study*—If applicable, explain how matching of cases and controls was addressed  *Cross-sectional study*—If applicable, describe analytical methods taking account of sampling strategy | 8 & 9, lines 251-274 |
|  |  | (*e*) Describe any sensitivity analyses |  |

Continued on next page

| Results | | | |
| --- | --- | --- | --- |
| Participants | 13* | (a) Report numbers of individuals at each stage of study—eg numbers potentially eligible, examined for eligibility, confirmed eligible, included in the study, completing follow-up, and analysed | 9-11, lines 265- 334 |
|  |  | (b) Give reasons for non-participation at each stage | 9-11, lines 265- 334 |
|  |  | (c) Consider use of a flow diagram | 9, line 284 |
| Descriptive data | 14* | (a) Give characteristics of study participants (eg demographic, clinical, social) and information on exposures and potential confounders | 9, lines 276-284 |
|  |  | (b) Indicate number of participants with missing data for each variable of interest | 9-11, lines 265- 334 |
|  |  | (c) *Cohort study*—Summarise follow-up time (eg, average and total amount) | - |
| Outcome data | 15* | *Cohort study*—Report numbers of outcome events or summary measures over time | *-* |
|  |  | *Case-control study—*Report numbers in each exposure category, or summary measures of exposure | *-* |
|  |  | Cross-sectional study—Report numbers of outcome events or summary measures | 9-11, lines 265- 334 |
| Main results | 16 | (*a*) Give unadjusted estimates and, if applicable, confounder-adjusted estimates and their precision (eg, 95% confidence interval). Make clear which confounders were adjusted for and why they were included | 9-11, lines 265- 334 |
|  |  | (*b*) Report category boundaries when continuous variables were categorized | 9-11, lines 265- 334 |
|  |  | (*c*) If relevant, consider translating estimates of relative risk into absolute risk for a meaningful time period | - |
| Other analyses | 17 | Report other analyses done—eg analyses of subgroups and interactions, and sensitivity analyses | - |
| Discussion | | | |
| Key results | 18 | Summarise key results with reference to study objectives | 11-15, lines 336-443 |
| Limitations | 19 | Discuss limitations of the study, taking into account sources of potential bias or imprecision. Discuss both direction and magnitude of any potential bias | 14, Lines 425-436 |
| Interpretation | 20 | Give a cautious overall interpretation of results considering objectives, limitations, multiplicity of analyses, results from similar studies, and other relevant evidence | 11-15, lines 336-443 |
| Generalisability | 21 | Discuss the generalisability (external validity) of the study results | 14, Lines 425-436 |
| Other information | | | |
| Funding | 22 | Give the source of funding and the role of the funders for the present study and, if applicable, for the original study on which the present article is based | Title Page &line 456-457 |

*Give information separately for cases and controls in case-control studies and, if applicable, for exposed and unexposed groups in cohort and cross-sectional studies.

**Note:** An Explanation and Elaboration article discusses each checklist item and gives methodological background and published examples of transparent reporting. The STROBE checklist is best used in conjunction with this article (freely available on the Web sites of PLoS Medicine at http://www.plosmedicine.org/, Annals of Internal Medicine at http://www.annals.org/, and Epidemiology at http://www.epidem.com/). Information on the STROBE Initiative is available at www.strobe-statement.org.
